# Prognostic value of androgen receptor and FOXA1 co-expression in non-metastatic triple negative breast cancer and correlation with other biomarkers

**DOI:** 10.1038/s41416-018-0142-6

**Published:** 2018-06-08

**Authors:** Séverine Guiu, Caroline Mollevi, Céline Charon-Barra, Florence Boissière, Evelyne Crapez, Elodie Chartron, Pierre-Jean Lamy, Marian Gutowski, Céline Bourgier, Gilles Romieu, Joelle Simony-Lafontaine, William Jacot

**Affiliations:** 1 Department of Medical Oncology, Montpellier Cancer Institute, Montpellier, France; 2 IRCM INSERM Unit 1194, University of Montpellier, Montpellier, France; 3 Biometry Department, Montpellier Cancer Institute, Montpellier, France; 4 Department of Pathology, Centre Georges-François Leclerc, Dijon, France; 5 Translational Research Unit, Montpellier Cancer Institute, Montpellier, France; 6 Department of surgery, Montpellier Cancer Institute, Montpellier, France; 7 Department of radiotherapy, Montpellier Cancer Institute, Montpellier, France

**Keywords:** Breast cancer, Prognostic markers

## Abstract

**Background:**

In luminal androgen receptor (AR) tumours, FOXA1 may direct AR to sites occupied by ER in luminal tumours, thus stimulating proliferation.

**Methods:**

AR and FOXA1 expression were evaluated by immunohistochemistry in 333 non-metastatic triple-negative breast cancers (TNBC). Positivity threshold was set at ≥ 1% staining. Lymphocytic infiltration, PD-L1expression, *PIK3CA* mutations, PTEN defects and *BRCA1* promoter methylation were assessed.

**Results:**

AR + /FOXA1 + tumours (42.4%) were more frequently: found in older patients, lobular, of lower nuclear grade, with more frequently *PIK3CA* mutations; exhibited less frequently *BRCA1* promoter methylation, defects of PTEN and PD-L1 expression than others. Recurrence-free and overall survivals were significantly lower for AR + /FOXA1 + TNBC (median follow-up: 7.8 years).

**Conclusions:**

AR + /FOXA1 + expression defines a luminal-like TNBC subgroup affected with a worse outcome compared to other TNBC and a higher risk of late recurrences. This subgroup appears enriched in *PIK3CA* mutations, suggesting a role for PI3K inhibitors in this subgroup.

## Introduction

Triple negative breast cancers (TNBCs) represent 15% of all breast cancer (BC) and are defined by the lack of oestrogen receptor (ER), progesterone receptor (PR) and HER2 expression/amplification. This subgroup of BC is extremely heterogeneous: Lehmann et al. distinguished seven TNBC subtypes displaying unique gene expression profiles, including the luminal androgen receptor (LAR) subtype.^1^ These tumours are ER-, CK5/6-, but express genes usually expressed in ER + luminal tumours such as the *androgen receptor* (AR) and *FOXA1*, are distinct of basal-like tumours and represent 8–12% of all BC. LAR cell lines are sensitive to AR antagonists.^1^ Using immunohistochemistry (IHC), AR is expressed in 8–53% of TNBC^2^ and its prognostic value is controversial.^3, 4^

The phosphatidylinositol 3-kinase (PI3K) signalling pathway is crucial for cell growth and survival. *PIK3CA* activating mutations and PTEN loss of expression may contribute to BC therapeutic resistance. In the study of Lehmann et al., *PIK3CA* mutations were more frequent in AR + TNBC than in AR- TNBC.^5^

Programmed cell death (PD-1) is an immune checkpoint receptor. Its ligand, PD-L1, is expressed by immune cells, BC cells, and tumour-infiltrating lymphocytes (TILs). PD-1 binding to PD-L1 specifically inhibits T-cell activation and represents one mechanism of immune tumour escape.^6^ TNBCs are thought to be more immunogenic than other BC and AR + TNBC show a higher frequency of PD-L1 expression.^7^

In LAR tumours, AR functionality is dependent on FOXA1: FOXA1 is required for AR binding chromatin, AR transcriptional activity and cell growth, directing AR to sites normally occupied by ER in luminal tumours, inducing an oestrogen-like programme stimulating proliferation.^8^ Our team has shown that AR + /FOXA1 + TNBC even tend to behave like luminal tumours.^9^ These two biomarkers could be useful to identify a particular subgroup of TNBC.

This study aimed to evaluate in a large series of non-metastatic TNBC with a long follow-up both the profiles and the prognostic value of AR/FOXA1 co-expression and its correlation with other biomarkers like PD-L1 and *PIK3*CA status.

## Materials and methods

This study included 349 patients with unifocal, unilateral, untreated, non-metastatic TNBC operated in our institution between 2002 and 2012. ER and PR negativity was defined as < 10% by IHC and HER2 negativity was defined as IHC 0/1 + or 2 + and negative fluorescent/chromogenic hybridisation in situ.

TMAs construction and immunostainings are described in Supplemental data 1. Immunostainings not evaluable on TMAs were assessed in full face sections. TMA sections were analysed independently by two trained observers both blinded to the clinicopathological characteristics and patient outcomes. Clashing cases were revised by a third observer to reach consensus. Results from the duplicate cores, when available, were averaged. A basal-like phenotype was defined by CK5/6 + and/or EGFR + ( > 10% tumour cells). As absence or reduced BRCA1 expression is linked to *BRCA1* promoter hypermethylation and is associated with basal markers, we also assessed *BRCA1* promoter methylation status. AR and FOXA1 positivity cut-off was ≥ 1% (nuclear staining) (Supplemental Fig. 1). We evaluated TILs (both peritumoural and intratumoural) on HES-stained sections. TILs density was scored as: 0 (no TILs), 1 (rare TILs), 2 (moderate infiltrate, less TILs than tumour cells), 3 (diffuse infiltrate, more TILs than tumour cells). PD-1 and PD-L1 expression by TILs was scored as follows: non-evaluable (NE, no TILs), 0 (no stained TIL), 1 ( < 10% of stained TILs), 2 (10–50% of stained TILs) and 3 ( > 50% of stained TILs). PD-L1 expression (IHC) by tumour cells was positive if ≥ 1%. Methods for DNA extraction, *PIK3CA* mutation detection, *PTEN* sequence copy number variations detection and *BRCA1* promoter methylation status are described in Supplemental data 2.

Statistical analyses are described in Supplemental data 3.

## Results

### AR expression

AR expression was available for 333 tumours. The concordance rate between the two cores was 85.1%. Patients with AR + tumours (58.6%, 195 tumours) were significantly older (*p* = 0.007), with a more frequent nodal involvement (40% vs 29%; *p* = 0.040) compared to AR- tumours. They exhibited tumours with significantly lower grades (grade 1-2: 32% vs 12.1%; *p* < 0.001), more frequent lobular histology (8.9% vs 1.5%; *p* = 0.007) and *PIK3CA* mutations (27.6% vs 3.3%; *p* < 0.001), less basal-like phenotype (53.6% vs 73%; *p* < 0.001), *BRCA1* promoter methylation (9.2% vs 35.9%; *p* < 0.001) and defects in PTEN (15.3% vs 34.1%; *p* = 0.009). There was no significant difference for TILs density, neither for PD-L1 expression (whatever the localisation) (Supplemental table 1).

### AR/FOXA1 co-expression

FOXA1 expression was available in 306 patients, including 185 patients (60.5%) with FOXA1 + tumour. The concordance rate between the two cores was 89.8%. Supplemental table 2 reports the clinical and tumour characteristics depending on the FOXA1 status. 42.4% patients had AR + /FOXA1 + tumours, 48 (15.8%) had AR + /FOXA1- tumours and 127 (41.8%) had AR- tumours. Patients with AR + /FOXA1 + tumours were significantly older (*p* = 0.003) than others. They exhibited tumours with significantly lower grades (grade 1-2: 38.3% vs 4.2% for AR + /FOXA1- vs 9.8% for AR-; *p* < 0.001), a more frequent lobular histology (10.2% vs 0% for AR + /FOXA1- vs 1.6% for AR-; *p* = 0.001) and *PIK3CA* mutations (*p* < 0.001; exon 9: 12.7%, exon 20: 19.1%), a lower frequency of basal-like phenotype (48.8% vs 66.7% for AR + /FOXA1- vs 74.0% for AR-; *p* < 0.001), *BRCA1* promoter methylation (4.8% vs 25.0% for AR + /FOXA1- vs 35.6% for AR-; *p* < 0.001), PTEN defects (*p* = 0.026) and PD-L1 expression [for both tumour cell membrane (*p* *=* 0.009) or TIL staining (*p* = 0.015)]. There was no significant difference regarding TILs density. AR + /FOXA1- and AR- tumours more frequently exhibited a staining of > 50% of TILs, compared to AR + /FOXA1 + tumours (10–50% of stained TILs more frequent) (Supplemental table 3).

### Survival analyses

After a 7.8 year median follow-up [0.6-14.7], 77 relapses [5-year recurrence-free survival (RFS) rate: 75.6%; 95%CI: 70.1-80.2] and 89 deaths [5-year overall survival (OS) rate: 81.7%; 95%CI: 76.9-85.6] were reported (Supplemental Figure 2).

**Fig. 1:**
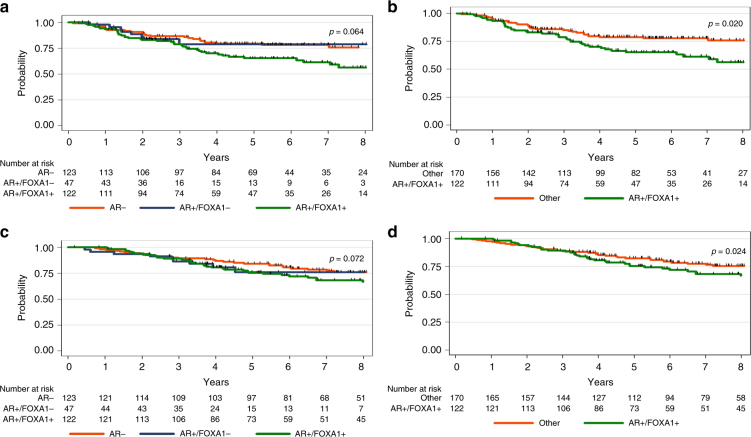
Relapse-free and overall survival according to both AR and FOXA1 statuses: **a**. RFS: Three subgroups AR + /FOXA1 + ; AR + /FOXA1-; AR-. **b**. RFS: AR + /FOXA1 + vs all other subgroups. **c**. OS: Three subgroups AR + /FOXA1 + ; AR + /FOXA1-; AR- tumours. **d**. OS: AR + /FOXA1 + vs all other subgroups

Patients with AR + /FOXA1 + , AR + /FOXA1- and AR- tumours showed 3-year RFS rates of 79.8%, 79.1% and 86.9% respectively and 5-year RFS rates of 66.8%, 79.1% and 79.7% (Fig. 1a). RFS was significantly shorter for AR + /FOXA1 + tumours compared with other tumours (AR + /FOXA1- and AR-) (*p* = 0.020) (Fig. 1b). Univariate analysis showed a significant association between RFS and tumour size (*p* < 0.001), nodal involvement (*p* < 0.001), adjuvant chemotherapy (*p* = 0.005), AR status (*p* = 0.034), AR/FOXA1 co-expression (*p* = 0.020), TILs density (*p* = 0.002) and PD-L1 expression by TILs (*p* = 0.018) (Supplemental table 4). In multivariate analysis, tumour size and nodal involvement were independent poor prognostic factors while adjuvant chemotherapy and TILs density were associated with a longer RFS (Supplemental table 5).

Five-years OS rates were 76.6%, 76.4% and 84.8% for patients with AR + /FOXA1 + , AR + /FOXA1- and AR- tumours, respectively (Fig. 1c). Patients with AR + /FOXA1 + tumours had a significantly worse OS (*p* = 0.024) (Fig. 1d). In univariate analysis, OS was significantly associated with age (*p* = 0.007), tumour size (*p* < 0.001), nodal involvement (*p* < 0.001), adjuvant chemotherapy (*p* < 0.001), AR status (*p* = 0.046), AR/FOXA1 co-expression (*p* = 0.024) and TILs density (*p* = 0.023) (Supplemental table 3). Tumour size, nodal involvement and AR/FOXA1 co-expression were found independent poor prognostic factors in multivariate analysis, while lobular histology, adjuvant chemotherapy and TILs density were associated with a longer OS (Supplemental table 5).

Among patients with a PD-L1 + tumour (*N* = 145), RFS (*p* = 0.012) and OS (*p* = 0.002) were shorter in case of AR/FOXA1 co-expression (Supplemental figure 3).

## Discussion

In our study, with cut-offs of 10% and 1% for ER/PR and AR positivity, respectively, we showed a AR + TNBC rate of 58.6%. There is currently no standardised assay to assess AR expression but recent data suggested that AR-targeted therapies may enhance the efficacy of chemotherapy even in TNBC with low AR expression by targeting cancer stem cell-like cells.^10^ In a meta-analysis of TNBC based on retrospective studies and population data, AR positivity was significantly associated with prolonged RFS but had no significant impact on OS.^4^ In our study, patients with AR + tumours had a poorer prognosis but AR was not an independent prognostic factor in multivariate analysis. Prospective data are needed to conclude on the independent prognostic value of this biomarker.

LAR tumours are frequently classified as luminal A or B but rarely as basal-like tumours by gene expression profiling.^1^ In a previous study, we found that AR + /FOXA1 + TNBC seemed to behave like luminal tumours with a morphological profile distinct from other TNBC, including AR + /FOXA1- tumours.^9^ In this independent series, we confirmed these data and added the notion of a lower frequency of basal-like phenotype and *BRCA1* promoter methylation in this population. The association of these two biomarkers easily identifies, in IHC, a particular subgroup of TNBC. In our study, patients with AR + /FOXA1 + tumours had a shorter RFS and OS than other TNBC and AR/FOXA1 co-expression was an independent prognostic factor for OS. Usually, in TNBC, relapses occur in the first 3 years of follow-up. These data are confirmed in our population for patients with AR- or AR + /FOXA1- tumours. Interestingly, in our study, relapses could occur even after a long follow-up in patients with AR + /FOXA1 + tumours, like in patients with ER + /HER2- tumours. Anti-androgen therapies are under development in AR + TNBC. For AR + /FOXA1 + TNBC with a risk of late relapse, an adjuvant anti-androgen therapy could be considered, like in ER + tumours.

Regarding the *PIK3CA* mutation rate (15.1%), our results are in accordance with the literature.^11^ Lehmann *et al* showed that *PIK3CA* mutations were significantly enriched among LAR TNBCs (46.2%) compared to all other TNBC subtypes (4.5%), with a predominance of exon 20 mutations that encode the catalytic domain of PI3K.^5^ We observed similar results with 27.7% of *PIK3CA* mutations in AR + TNBC (16.1% on exon 20) compared to 3.3% in AR- tumours (all on exon 9). PTEN inactivation leads to constitutive AKT activation, promoting cell growth, proliferation, survival, and migration. Millis *et al* reported 58% of PIK3CA or PTEN mutation/protein loss in AR + TNBC,^11^ which is concordant with our results (40.0% *PIK3CA* mutation/PTEN aberration). Subgroup analysis showed a significantly higher *PIK3CA* mutation rate in the AR + /FOXA1 + subgroup (31.8% vs 16.7% in AR + /FOXA1- and 3.5% in AR-). Ongoing clinical trials are currently evaluating the association of anti-androgen therapy and PIK3 inhibitor. FOXA1 will allow the identification of a TNBC subgroup that could retrieve more benefit from this therapeutic strategy.

Stromal TILs constitute a robust and an independent prognostic marker in TNBC treated with chemotherapy. In our series, TILs density was also a strong and independent prognostic factor for both RFS and OS. To the best of our knowledge, no study has yet evaluated the correlation between TILs and AR expression. In our study, there was no difference between AR + and AR- tumours on this point, including in the subgroup analyses according to FOXA1 status.

Cancer cell could escape from immune surveillance by upregulating PD-L1 expression. The rate of PD-L1 expression in TNBC is extremely variable across studies, due to the lack of standard assay and its prognostic value remains controversial. In our study, 56.1% of TNBC expressed PD-L1 and it was not an independent prognostic marker in multivariate analysis. The 1% threshold for positivity was selected based on data demonstrating a clinical response to PD-L1 inhibition at this expression level in some cancers. There was no difference regarding PD-L1 expression and AR status in our series, contrary to the results of Tung et al.^7^ reporting a 2.6-fold higher PD-L1 rate in their AR + TNBC population.^7^ A higher rate of PD-L1 expression was seen in the AR + /FOXA1- subgroup (76.7% vs 49.6% in AR + /FOXA1 + and 55.2% in AR-). Among patients with a PD-L1 + tumour, we found significantly poorer RFS and OS in case of AR/FOXA1 co-expression. It could be interesting to specifically evaluate the benefit of anti-PD1 or PD-L1 targeted therapies in association with an anti-androgen in this subgroup.

In this large homogeneous series with a long follow-up, 42% of non-metastatic TNBC presented an AR/FOXA1 co-expression. AR + /FOXA1 + expression defines a luminal-like TNBC subgroup with a worse outcome compared to other TNBC and a higher risk of late recurrences mimicking luminal tumours behaviour and suggesting a putative role for anti-androgen therapies. This subgroup appears enriched in *PIK3CA* mutations, advocating for PIK3 inhibitors evaluation, alone or in association with anti-androgens, in this specific subgroup. All these data suggest the need for a concomitant evaluation of AR/FOXA1 to identify a specific subgroup of patients with TNBC who could benefit from targeted therapies.

## Electronic supplementary material

Supplemental Figure 1

Supplemental Figure 2

Supplemental Figure 3A

Supplemental Figure 3B

Supplemental Table 1

Supplemental Table 2

Supplemental Table 3

Supplemental Table 4

Supplemental Table 5

Supplemental data 1

## Availability of data and materials

Materials described in the study are available in the Translational Research Unit, Montpellier Cancer Institute, Montpellier, France. Data are available in the Biometry Department, Montpellier Cancer Institute, Montpellier, France.
