# Supplementary figures and images for: Prognostic value of androgen receptor and FOXA1 co-expression in non-metastatic triple negative breast cancer and correlation with other biomarkers
Source: Br J Cancer. 2018 Jun 8;119(1):76–9. doi: 10.1038/s41416-018-0142-6 (PMC6035246; doi:10.1038/s41416-018-0142-6)

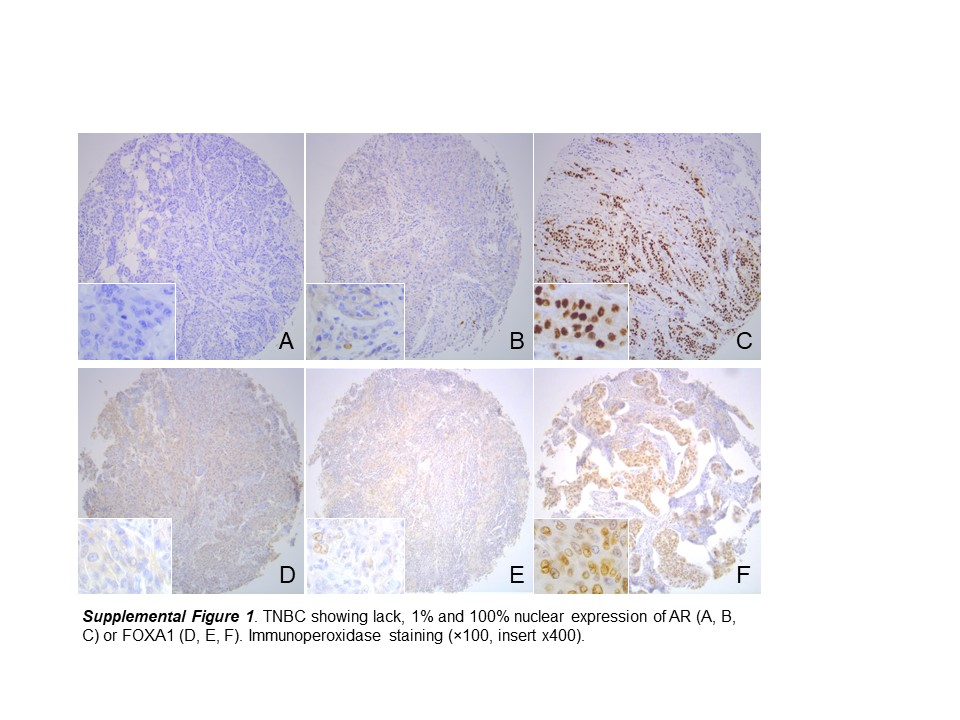

Supplement: Supplementary file 1 — Supplemental Figure 1 [file 41416_2018_142_MOESM1_ESM.tif]

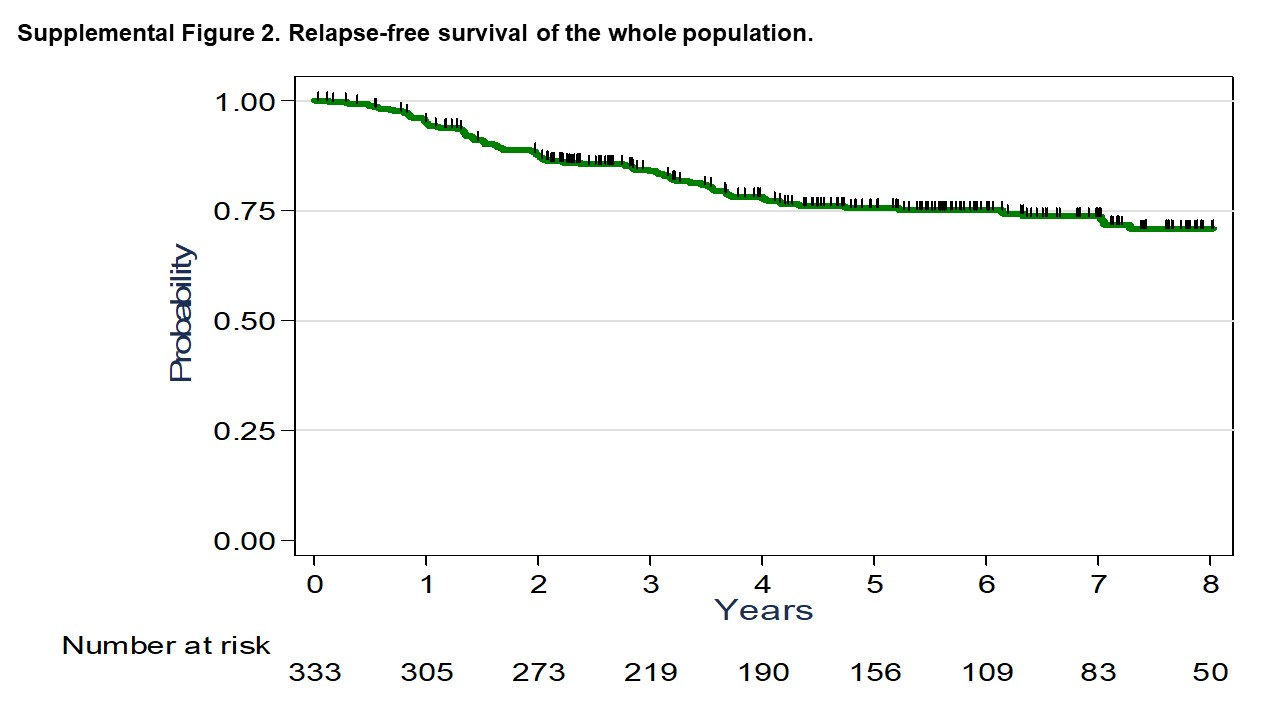

Supplement: Supplementary file 2 — Supplemental Figure 2 [file 41416_2018_142_MOESM2_ESM.tif]

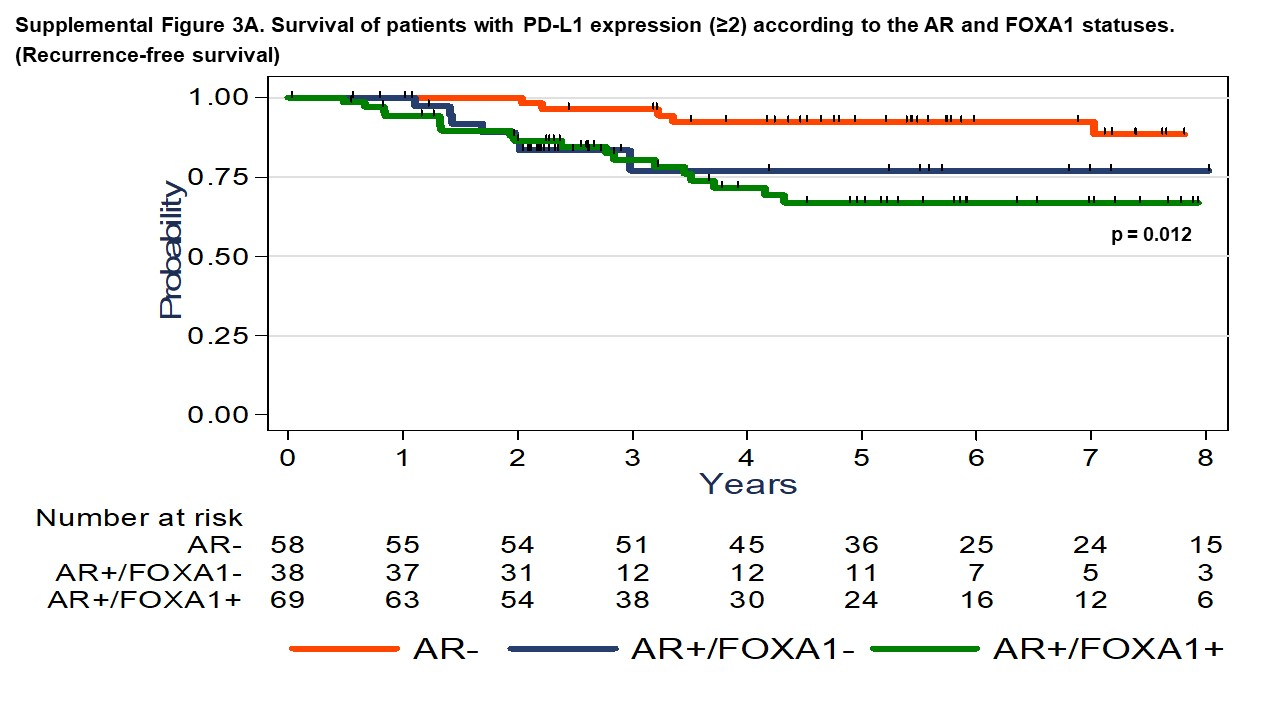

Supplement: Supplementary file 3 — Supplemental Figure 3A [file 41416_2018_142_MOESM3_ESM.tif]

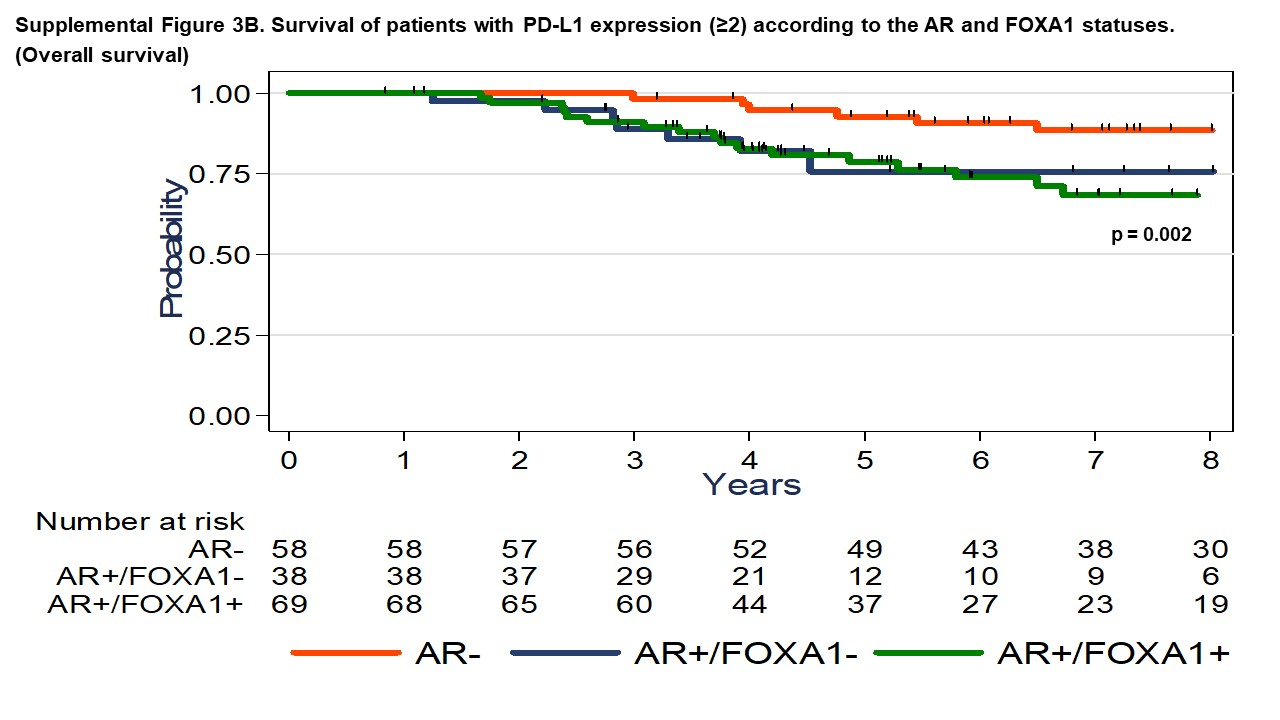

Supplement: Supplementary file 4 — Supplemental Figure 3B [file 41416_2018_142_MOESM4_ESM.tif]
