# Supplementary material for: Prognostic value of androgen receptor and FOXA1 co-expression in non-metastatic triple negative breast cancer and correlation with other biomarkers
Source: Br J Cancer. 2018 Jun 8;119(1):76–9. doi: 10.1038/s41416-018-0142-6 (PMC6035246; doi:10.1038/s41416-018-0142-6)
Supplement: Supplementary file 5 — Supplemental Table 1 [file 41416_2018_142_MOESM5_ESM.docx]

**Supplemental Table 1. Clinicopathological characteristics of the whole population and of patients with AR positive and AR negative tumors**

|  | **Overall population**  **N=333** | **AR positive**  **N=195 (58.6%)** | **AR negative**  **N=138 (41.4%)** | ***P value*** |
| --- | --- | --- | --- | --- |
| **Age (*years*),** median [min-max]  *< 55 years*  *≥ 55 years* | 58.1 [28.5-98.6]  143 (42.9%)  190 (57.1%) | 59.1 [28.7-98.6]  74 (38%)  121 (62%) | 55.0 [28.5-87.2]  69 (50%)  69 (50%) | **0.007**  **0.030** |
| **Tumor size**  T1  T2  T3/T4 | 154 (46.4%)  156 (47.0%)  22 (6.6%) | 94 (48.2%)  88 (45.1%)  13 (6.7%) | 60 (43.8%)  68 (49.6%)  9 (6.6%) | 0.710 |
| **Nodal status**  N-  N+ | 215 (64.6%)  118 (35.4%) | 117 (60%)  78 (40%) | 98 (71%)  40 (29%) | **0.040** |
| **Histological grade (SBR)**  1-2  3 | 78 (23.9%)  248 (76.1%) | 62 (32%)  132 (68%) | 16 (12.1%)  116 (87.9%) | **<0.001** |
| **Histology**  Ductal  Lobular  Other | 270 (81.8%)  19 (5.8%)  41 (12.4%) | 155 (80.7%)  17 (8.9%)  20 (10.4%) | 115 (83.3%)  2 (1.5%)  21 (15.2%) | **0.007** |
| **Adjuvant chemotherapy**  No  Yes | 88 (26.6%)  243 (73.4%) | 58 (29.9%)  136 (70.1%) | 30 (21.9%)  107 (78.1%) | 0.105 |
| **Basal-like phenotype**  Yes  No  Missing | 203 (61.7%)  126 (38.3%)  4 | 103 (53.6%)  89 (46.4%)  3 | 100 (73%)  37 (27%)  1 | **<0.001** |
| **BRCA1 promoter methylation**  Yes  No  Missing | 41 (22.9%)  138 (77.1%)  154 | 8 (9.2%)  79 (90.8%)  108 | 33 (35.9%)  59 (64.1%)  46 | **<0.001** |
| ***PIK3CA* mutations**  None  Exon 9  Exon 20  Missing | 152 (84.9%)  13 (7.3%)  14 (7.8%)  154 | 63 (72.4%)  10 (11.5%)  14 (16.1%)  108 | 89 (96.7%)  3 (3.3%)  0  46 | **<0.001** |
| **PTEN status**  Normal  Deletion  Amplification  Missing | 132 (75%)  40 (22.7%)  4 (2.3%)  157 | 72 (84.7%)  12 (14.1%)  1 (1.2%)  110 | 60 (65.9%)  28 (30.8%)  3 (3.3%)  47 | **0.009** |
| **TILs density**  [0-2]  3  Missing | 230 (75.2%)  76 (24.8%)  27 | 134 (75.7%)  43 (24.3%)  18 | 96 (74.4%)  33 (25.6%)  9 | 0.800 |
| **PD-L1 expression tumor cells**  < 1%  ≥ 1%  Missing | 129 (43.9%)  165 (56.1%)  39 | 72 (43.6%)  93 (56.4%)  30 | 57 (44.2%)  72 (55.8%)  9 | 0.925 |
| **PD-L1 expression TILs**  0  ]0-10]  ]10-50]  >50  Missing | 52 (17.9%)  94 (32.3%)  81 (27.8%)  64 (22.0%)  42 | 25 (15.2%)  50 (30.5%)  48 (29.3%)  41 (25.0%)  31 | 27 (21.3%)  44 (34.6%)  33 (26.0%)  23 (18.1%)  11 | 0.301 |
| **PD-1 expression TILs**  0  ]0-10]  ]10-50]  >50  Missing | 78 (26.1%)  76 (25.4%)  120 (40.1%)  25 (8.4%)  34 | 48 (27.9%)  35 (20.4%)  79 (45.9%)  10 (5.8%)  23 | 30 (23.6%)  41 (32.3%)  41 (32.3%)  15 (11.8%)  11 | **0.011** |
| AR: Androgen Receptor; SBR: Scarff-Bloom-Richardson system; TILs: Tumor-infiltrating Lymphocytes; PD-1: Programmed cell death1; PD-L1: Programmed cell death ligand 1 | | | | |
