# Supplementary material for: Prognostic value of androgen receptor and FOXA1 co-expression in non-metastatic triple negative breast cancer and correlation with other biomarkers
Source: Br J Cancer. 2018 Jun 8;119(1):76–9. doi: 10.1038/s41416-018-0142-6 (PMC6035246; doi:10.1038/s41416-018-0142-6)
Supplement: Supplementary file 6 — Supplemental Table 2 [file 41416_2018_142_MOESM6_ESM.docx]

**Supplemental Table 2. Clinicopathological characteristics of the whole population and of patients with FOXA1 positive and FOXA1 negative tumors**

|  | **Overall population**  **N=306** | **FOXA1 positive**  **N=185 (60.5%)** | **FOXA1 negative**  **N=121 (39.5%)** | ***P value*** |
| --- | --- | --- | --- | --- |
| **Age (*years*),** median [min-max]  *< 55y*  *≥ 55y* | 57.5 [28.5-98.6]  138 (45.1%)  168 (54.9%) | 58.4 [28.5-89.1]  76 (41.1%)  109 (58.9%) | 54.8 [28.7-98.6]  62 (51.2%)  59 (48.8%) | 0.060  0.081 |
| **Tumor size**  T1  T2  T3/T4 | 140 (45.9%)  142 (46.6%)  23 (7.5%) | 90 (48.9%)  80 (43.5%)  14 (7.6%) | 50 (41.3 %)  62 (51.2%)  9 (7.4%) | 0.394 |
| **Nodal status**  N-  N+ | 197 (64.4%)  109 (35.6%) | 115 (62.2%)  70 (37.8%) | 82 (67.8%)  39 (32.2%) | 0.317 |
| **Histological grade (SBR)**  1-2  3 | 64 (21.3%)  236 (78.7%) | 55 (30.2%)  127 (69.8%) | 9 (7.6%)  109 (92.4%) | **<0.001** |
| **Histology**  Ductal  Lobular  Other | 251 (82.8%)  15 (5.0%)  37 (12.2%) | 142 (77.6%)  14 (7.6%)  27 (14.8%) | 109 (90.3%)  1 (0.8%)  10 (8.3%) | **0.005** |
| **Adjuvant chemotherapy**  No  Yes | 76 (25.0%)  228 (75.0%) | 45 (24.5%)  139 (75.5%) | 31 (25.8%)  89 (74.2%) | 0.786 |
| **Basal-like phenotype**  Yes  No  Missing | 189 (62.4%)  114 (37.6%)  3 | 104 (57.1%)  78 (42.9%)  3 | 85 (70.3%)  36 (29.7%)  0 | **0.021** |
| **BRCA1 promoter methylation**  Yes  No  Missing | 37 (22.7%)  126 (77.3%)  143 | 13 (13.4%)  84 (86.6%)  88 | 24 (36.4%)  42 (63.6%)  55 | **<0.001** |
| ***PIK3CA* mutations**  None  Exon 9  Exon 20  Missing | 138 (84.7%)  11 (6.8%)  14 (8.6%)  143 | 77 (79.4%)  8 (8.3%)  12 (12.4%)  88 | 61 (92.4%)  3 (4.6%)  2 (3.0%)  55 | **0.062** |
| **PTEN status**  Normal  Deletion  Amplification  Missing | 118 (73.8%)  38 (23.7%)  4 (2.5%)  146 | 76 (80.9%)  16 (17.0%)  2 (2.1%)  91 | 42 (63.7%)  22 (33.3%)  2 (3.0%)  55 | **0.049** |
| **TILs density**  [0-2]  >2  Missing | 223 (75.3%)  73 (24.7%)  10 | 134 (75.3%)  44 (24.7%)  7 | 89 (75.4%)  29 (25.6%)  3 | 0.978 |
| **PD-L1 expression tumor cells**  < 1%  ≥ 1%  Missing | 124 (43.8%)  159 (56.2%)  23 | 82 (48.8%)  86 (51.2%)  17 | 42 (36.5%)  73 (63.5%)  6 | **0.041** |
| **PD-L1 expression TILs**  0  1  2  3  Missing | 49 (17.5%)  90 (32.1%)  78 (27.9%)  63 (22.5%)  26 | 33 (19.9%)  55 (33.1%)  46 (27.7%)  32 (19.3 %)  19 | 16 (14.0%)  35 (30.7%)  32 (28.1%)  31 (27.2%)  7 | 0.344 |
| **PD-1 expression TILs**  0  1  2  3  Missing | 73 (25.2%)  75 (25.9%)  118 (40.7%)  24 (8.3%)  16 | 49 (28.2%)  39 (22.4%)  77 (44.2%)  9 (5.2%)  11 | 24 (20.7%)  36 (31.0%)  41 (35.4 %)  15 (12.9%)  5 | **0.019** |

SBR: Scarff-Bloom-Richardson system; TILs: Tumor-infiltrating Lymphocytes; PD-1: Programmed cell death1; PD-L1: Programmed cell death ligand 1
