# Supplementary material for: Prognostic value of androgen receptor and FOXA1 co-expression in non-metastatic triple negative breast cancer and correlation with other biomarkers
Source: Br J Cancer. 2018 Jun 8;119(1):76–9. doi: 10.1038/s41416-018-0142-6 (PMC6035246; doi:10.1038/s41416-018-0142-6)
Supplement: Supplementary file 7 — Supplemental Table 3 [file 41416_2018_142_MOESM7_ESM.docx]

**Supplemental Table 3. Clinicopathological characteristics of patients with AR+/FOXA1+, AR+/FOXA1- and AR negative tumors.**

|  | **AR+/FOXA1+**  **N=129 (42.4%)** | **AR+/FOXA1-**  **N=48 (15.8%)** | **AR negative**  **N=127 (41.8%)** | ***P value*** |
| --- | --- | --- | --- | --- |
| **Age (*years*),** median [min-max]  *< 55 years*  *≥ 55 years* | 59.5 [36.6-89.1]  46 (35.7%)  83 (64.3%) | 54.2 [28.7-98.6]  25 (52.1%)  23 (47.9%) | 54.6 [28.5-86.3]  66 (52.0%)  61 (48.0%) | **0.003**  **0.018** |
| **Tumor size**  T1  T2  T3/T4 | 64 (49.6%)  57 (44.2%)  8 (6.2%) | 23 (47.9%)  21 (43.8%)  4 (8.3%) | 53 (42.1%)  64 (50.8%)  9 (7.1%) | 0.756 |
| **Nodal status**  N-  N+ | 77 (59.7%)  52 (40.3%) | 30 (62.5%)  18 (37.5%) | 90 (70.9%)  37 (29.1%) | 0.162 |
| **Histological grade (SBR)**  1-2  3 | 49 (38.3%)  79 (61.7%) | 2 (4.2%)  46 (95.8%) | 12 (9.8%)  110 (90.2%) | **<0.001** |
| **Histology**  Ductal  Lobular  Other | 97 (76.4%)  13 (10.2%)  17 (13.4%) | 46 (97.9%)  0 (0%)  1 (2.1%) | 107 (84.2%)  2 (1.6%)  18 (14.2%) | **0.001** |
| **Adjuvant chemotherapy**  No  Yes | 38 (29.7%)  90 (70.3%) | 12 (25.0%)  36 (75.0%) | 26 (20.6%)  100 (79.4%) | 0.251 |
| **Basal-like phenotype**  Yes  No | 62 (48.8%)  65 (51.2%) | 32 (66.7%)  16 (33.3%) | 94 (74.0%)  33 (26.0%) | **<0.001** |
| **BRCA1 promoter methylation**  Yes  No  Missing | 3 (4.8%)  60 (95.2%)  66 | 3 (25.0%)  9 (75.0%)  36 | 31 (35.6%)  56 (64.4%)  40 | **<0.001** |
| ***PIK3CA* mutations**  None  Exon 9  Exon 20  Missing | 43 (68.2%)  8 (12.7%)  12 (19.1%)  66 | 10 (83.3%)  0 (0%)  2 (16.7%)  36 | 84 (96.5%)  3 (3.5%)  0 (0%)  40 | **<0.001** |
| **PTEN status**  Normal  Deletion  Amplification  Missing | 53 (86.9%)  7 (11.5%)  1 (1.6%)  68 | 8 (66.7%)  4 (33.3%)  0 (0%)  36 | 56 (65.1%)  27 (31.4%)  3 (3.5%)  41 | **0.026** |
| **TILs density**  [0-2]  3  Missing | 93 (75.6%)  30 (24.4%)  6 | 35 (74.5%)  12 (25.5%)  1 | 94 (75.2%)  31 (24.8%)  2 | 0.988 |
| **PD-L1 expression tumor cells**  < 1%  ≥ 1%  Missing | 58 (50.4%)  57 (49.6%)  14 | 10 (23.3%)  33 (76.7%)  5 | 56 (44.8%)  69 (55.2%)  2 | **0.009** |
| **PD-L1 expression TILs**  0  ]0-10]  ]10-50]  >50  Missing | 20 (17.5%)  40 (35.1%)  31 (27.2%)  23 (20.2%)  15 | 3 (7.0%)  7 (16.3%)  16 (37.2%)  17 (39.5%)  5 | 26 (21.1%)  43 (35.0%)  31 (25.2%)  23 (18.7%)  4 | **0.015** |
| **PD-1 expression TILs**  0  ]0-10]  ]10-50]  >50  Missing | 32 (26.7%)  26 (21.7%)  58 (48.3%)  4 (3.3%)  9 | 13 (28.3%)  7 (15.2%)  20 (43.5%)  6 (13.0%)  2 | 28 (22.8%)  41 (33.3%)  40 (32.5%)  14 (11.4%)  4 | **0.009** |

AR: Androgen Receptor; SBR: Scarff-Bloom-Richardson system; TILs: Tumor-infiltrating Lymphocytes; PD-1: Programmed cell death1; PD-L1: Programmed cell death ligand 1
