# Supplementary material for: Prognostic value of androgen receptor and FOXA1 co-expression in non-metastatic triple negative breast cancer and correlation with other biomarkers
Source: Br J Cancer. 2018 Jun 8;119(1):76–9. doi: 10.1038/s41416-018-0142-6 (PMC6035246; doi:10.1038/s41416-018-0142-6)
Supplement: Supplementary file 8 — Supplemental Table 4 [file 41416_2018_142_MOESM8_ESM.docx]

**Supplemental Table 4. Univariate analysis.**

|  | **N** | **RFS** | | | **OS** | | |
| --- | --- | --- | --- | --- | --- | --- | --- |
|  |  | **Events** | **5-y RFS** | **HR [95% CI]** | **Events** | **5-y OS** | **HR [95% CI]** |
| **Age (years)**  < 55  ≥ 55 | 143  190 | 26  51  *p* = 0.082 | 81.0  71.5 | 1  1.52 [0.95-2.43] | 28  61  ***p* = 0.007** | 88.1  77.0 | 1  1.82 [1.16-2.85] |
| **T**  T1  T2  T3/T4 | 154  156  22 | 20  45  12  ***p* < 0.001** | 85.9  70.3  45.0 | 1  2.30 [1.36-3.90]  5.30 [2.59-10.9] | 20  55  14  ***p* < 0.001** | 91.7  76.2  53.4 | 1  2.84 [1.71-4.75]  6.61 [3.33-13.1] |
| **N**  N-  N+ | 215  118 | 28  49  ***p* < 0.001** | 86.1  57.9 | 1  3.59 [2.26-5.71] | 41  48  ***p* < 0.001** | 89.9  67.2 | 1  2.4 [1.58-3.65] |
| **SBR**  1-2  3 | 78  248 | 18  59  *p* = 0.6 | 78.5  73.9 | 1  1.15 [0.68-1.95] | 23  65  *p* = 0.855 | 85.2  80.0 | 1  1.04 [0.65-1.68] |
| **Histology**  Ductal  Lobular  Other | 270  19  41 | 66  6  4  *p* = 0.081 | 73.9  72.6  88.9 | 1  1.21 [0.52-2.78]  0.35 [0.13-0.95] | 78  4  7  *p* = 0.140 | 79.2  89.5  92.6 | 1  0.68 [0.25-1.85]  0.48 [0.22-1.05] |
| **Adjuvant chemotherapy**  No  Yes | 88  243 | 30  47  ***p* = 0.005** | 63.9  80.0 | 1  0.53 [0.33-0.84] | 41  48  ***p* < 0.001** | 68.5  86.5 | 1  0.38 [0.25-0.58] |
| **AR status**  Negative  Positive | 138  195 | 26  51  ***p* = 0.034** | 81.4  70.6 | 1  1.66 [1.03-2.67] | 32  57  ***p* = 0.046** | 86.0  78.2 | 1  1.55 [1.00-2.39] |
| **FOXA1 status**  Negative  Positive | 121  183 | 26  48  *p* = 0.405 | 77.4  72.0 | 1  1.22 [0.76-1.97] | 30  58  *p* = 0.313 | 79.2  80.9 | 1  1.25 [0.80-1.95] |
| **AR/FOXA1 status**  Other  AR+/FOXA1+ | 175  129 | 34  40  ***p* = 0.020** | 79.3  66.8 | 1  1.71 [1.08-2.70] | 41  47  ***p* = 0.024** | 83.0  76.6 | 1  1.61 [1.06-2.45] |
| **TIL density**  [0, 2]  3 | 230  76 | 67  8  ***p* = 0.002** | 69  89.5 | 1  0.33 [0.16-0.69] | 75  13  ***p* = 0.023** | 76.6  92.2 | 1  0.51 [0.28-0.92] |
| **PD-L1 expression tumor cells**  < 1%  ≥ 1% | 129  165 | 40  32  *p* = 0.057 | 69.5  77.8 | 1  0.64 [0.40-1.02] | 43  40  *p* = 0.313 | 79.8  81.2 | 1  0.8 [0.52-1.23] |
| **PD-L1 expression TILs**  0  ]0-10]  ]10-50]  >50 | 52  94  81  64 | 13  32  9  14  ***p* = 0.018** | 71.0  68.4  89.6  72.6 | 1  1.33 [0.69-2.53]  0.43 [0.18-1.00]  0.86 [0.40-1.83] | 13  36  18  12  *p* = 0.154 | 83.6  73.9  84.9  88.8 | 1  1.42 [0.75-2.68]  0.85 [0.42-1.74]  0.77 [0.35-1.69] |
| **PD-1 expression TILs**  0  ]0-10]  ]10-50]  >50 | 78  76  120  25 | 19  21  28  6  *p* = 0.999 | 75.5  75.4  71.7  72.7 | 1  1.04 [0.56-1.94]  1.02 [0.57-1.83]  1.02 [0.41-2.56] | 23  22  34  8  *p* = 0.733 | 79.4  82.7  80.8  73.0 | 1  0.87 [0.49-1.57]  1.15 [0.68-1.96]  1.24 [0.55-2.78] |

RFS: Recurrence-free survival; OS: Overall survival; HR: Hazard Ratio; 95% CI: 95% Confidence Interval; SBR: Scarff-Bloom-Richardson system;
AR: Androgen Receptor; TILs: Tumor-infiltrating Lymphocytes; PD-1: Programmed cell death1; PD-L1: Programmed cell death ligand 1
