# Supplementary material for: Prognostic value of androgen receptor and FOXA1 co-expression in non-metastatic triple negative breast cancer and correlation with other biomarkers
Source: Br J Cancer. 2018 Jun 8;119(1):76–9. doi: 10.1038/s41416-018-0142-6 (PMC6035246; doi:10.1038/s41416-018-0142-6)
Supplement: Supplementary file 9 — Supplemental Table 5 [file 41416_2018_142_MOESM9_ESM.docx]

**Supplemental Table 5. Multivariate analysis.**

|  | **RFS (N = 303)** | | | **OS (N = 289)** | | |
| --- | --- | --- | --- | --- | --- | --- |
|  | **HR** | **95% CI** | ***P*** | **HR** | **95% CI** | ***p*** |
| **T**  T1  T2  T3-T4 | 1  1.92  1.91 | [1.11-3.31]  [0.87-4.20] | **0.045** | 1  2.40  4.03 | [1.41-4.09]  [1.84-8.83] | **< 0.001** |
| **N**  N-  N+ | 1  3.9 | [2.34-6.52] | **< 0.001** | 1  2.19 | [1.35-3.54] | **0.001** |
| **Histology**  Ductal  Lobular  Other |  |  |  | 1  0.27  0.49 | [0.08-0.91]  [0.23-1.09] | **0.011** |
| **Adjuvant chemotherapy**  No  Yes | 1  0.41 | [0.25-0.66] | **< 0.001** | 1  0.35 | [0.23-0.55] | **< 0.001** |
| **AR/FOXA1 status**  Other  AR+/FOXA1+ |  |  |  | 1  1.57 | [1.01-2.45] | **0.044** |
| **TIL density**  [0, 2]  3 | 1  0.35 | [0.16-0.72] | **0.001** | 1  0.53 | [0.29-0.96] | **0.026** |

RFS: Recurrence-free survival; OS: Overall survival; HR: Hazard Ratio; 95% CI: 95% Confidence Interval; AR: Androgen Receptor; TILs: Tumor-infiltrating Lymphocytes
