# Supplementary material for: Prognostic value of androgen receptor and FOXA1 co-expression in non-metastatic triple negative breast cancer and correlation with other biomarkers
Source: Br J Cancer. 2018 Jun 8;119(1):76–9. doi: 10.1038/s41416-018-0142-6 (PMC6035246; doi:10.1038/s41416-018-0142-6)
Supplement: Supplementary file 10 — Supplemental data 1 [file 41416_2018_142_MOESM10_ESM.docx]

**Supplemental data 1. Construction of TMAs and immunostainings**

Tissue blocks appearing to have enough material during gross inspection were initially selected from the Biological Resource Center of our Institute (Biobank number BB-0033-00059). Hematoxylin–eosin-safranin (HES)-stained sections were evaluated by a pathologist for the presence of carcinoma. Two representative tumor areas, to be used for the construction of the TMAs, were identified on each slide. The tissues corresponding to selected areas were sampled using a manual arraying instrument (Manual Tissue Arrayer 1, Beecher Instruments, Sun Prairie, WI, USA). The sampling consisted of two malignant cores (1 mm in diameter) from different areas of the tumor, placed at specified coordinates. When possible, normal breast epithelium was also selected as internal control. After the arraying was completed, 6 TMA blocks were sectioned at a thickness of 4 μm. One section was stained with HES and the others were used for IHC.

TMA sections were incubated with antibodies against cytokeratins 5/6 (mouse monoclonal, clone 6D5/16 B4, Dako), EGFR (mouse monoclonal, clone 31G7, inVitroGen), AR (mouse monoclonal, clone AR441, Dako), PD-1 (mouse monoclonal, clone MRQ-22, BioSB) and PD-L1 (rabbit monoclonal, clone SP142, Roche) on a Autostainer Link48 platform (Dako) using the Flex® system for signal amplification and diaminobenzidine tetrahydrochloride (DAB) as a chromogen. FoxA1 was detected with an indirect immunoperoxydase method using the goat polyclonal antibody HNF-3α/β (C-20) (Santa Cruz) on the Benchmark XT stainer (Ventana).

**Supplemental data 2. DNA extraction, *PIK3CA* mutation detection, PTEN sequence copy number variation detection and BRCA1 promoter methylation status**

DNA was extracted from frozen tumor tissue sample as previously described ([1](#_ENREF_1)) by using the QIAamp DNA Mini Kit (Qiagen GmbH, Hilden, Germany) according to the manufacturer’s instructions.

***PIK3CA* mutation detection**

Polymerase Chain Reaction (PCR) amplification and High Resolution Melting analysis were performed as previously described ([1](#_ENREF_1)) on a Rotor-Gene 6000™ instrument (Corbett Research, Mortlake, New South Wales, Australia) using the Light Cycler 480 High Resolution Melting (HRM) Master Mix kit (Roche Diagnostics, Meylan, France). Briefly, two primer pairs were designed to screen both the exon 9 and the exon 20 hotspot mutation regions. After HRM, sequence analysis of mutated profiles was performed using the Big Dye Terminator v1.1 kit (Applied Biosystems Inc., Foster City, CA) and the Applied Biosystems Sequencing Analysis® software v5.2.

**Detection of PTEN sequence copy number variations (CNV) by Multiplex Ligation-dependent Probe Amplification**

Multiplex Ligation-Dependent Probe Amplification (SALSA MLPA probemix P225-D1 PTEN, MRC-Holland, Amsterdam, the Netherlands) was carried out as previously described ([1](#_ENREF_1)) according to the manufacturer’s specification and interpretation was based on the use of the dedicated Coffalyser.Net software (MRC-Holland, Amsterdam, the Netherlands).

**BRCA1 promoter methylation status**

DNA methylation patterns at the CpG islands of the BRCA1 promoter were assessed using the previously described methylation-specific PCR assay ([2](#_ENREF_2)). Briefly, after DNA conversion by bisulphite treatment, PCR amplifications with two specific primer pairs (corresponding to the methylated or unmethylated BRCA1 DNA sequence) were performed in parallel ([2](#_ENREF_2)). Methylated and unmethylated amplicons were resolved through agarose gel electrophoresis.

1. Jacot W, Mollevi C, Fina F, Lopez-Crapez E, Martin PM, Colombo PE, et al. High EGFR protein expression and exon 9 PIK3CA mutations are independent prognostic factors in triple negative breast cancers. BMC cancer. 2015;15:986.

2. Esteller M, Silva JM, Dominguez G, Bonilla F, Matias-Guiu X, Lerma E, et al. Promoter hypermethylation and BRCA1 inactivation in sporadic breast and ovarian tumors. Journal of the National Cancer Institute. 2000;92(7):564-9.

**Supplemental data 3. Statistical analyses**

Descriptive analyses were performed using medians and ranges for continuous parameters, and frequencies and percentages for categorical parameters. Continuous parameters were compared using the Kruskal-Wallis test and categorical parameters using the chi-square test (or Fisher’s exact test, if appropriate). All tests were two-sided, and *p*-values less than 0.05 were considered statistically significant.

The median follow-up was calculated using the reverse Kaplan-Meier method. Recurrence-free survival (RFS) and overall survival (OS) were estimated using the Kaplan-Meier method and compared using the Log-rank test. RFS was defined as the time between the date of the first histology and the date of the breast cancer first recurrence at any site, or death from any cause. Surviving patients without recurrence and patients lost to follow-up were censored at the time of the last follow-up or last documented visit. OS was defined as the time between the date of the first histology and the date of death from any cause. Multivariate analyses were performed using the Cox proportional hazard model. Hazard ratios (HR) were given with their 95% confidence interval (95% CI). All statistical analyses were performed with the STATA 13.0 software (StatCorp, College Station, TX).
